# A BACKWARD CYCLING PROGRAMME FOR PEOPLE WITH PARKINSON’S DISEASE: A FEASIBILITY AND PRELIMINARY RESULTS STUDY

**DOI:** 10.2340/jrm.v56.17738

**Published:** 2024-06-11

**Authors:** Suzanne K. O’NEAL, Stephanie A. MILLER, Megan C. EIKENBERRY, Elizabeth S. MOORE

**Affiliations:** 1 Midwestern University, Glendale, AZ, USA; 2 University of Indianapolis, Indianapolis, IN, USA; 3 Marian University, Indianapolis, IN, USA

**Keywords:** exercise, Parkinson’s disease, postural balance, backward cycling

## Abstract

**Objective:**

To assess the feasibility of backward cycling for people with Parkinson’s disease. Secondary objectives were to assess changes in gait and balance following a 6-week program.

**Design:**

A single-group prospective pre-test, post-test study with 1-month follow-up.

**Subjects/Patients:**

Twenty-six people with Parkinson’s disease (mean age: 69 (7.74) years, gender: 83% males, time since diagnosis: 6 (4.44) years).

**Methods:**

Participants pedaled backward on a stationary bicycle for 30 minutes at moderate intensity twice a week for 6 weeks. Feasibility was assessed by acceptability, suitability, and burden. Data collected at pre- and post-intervention with 1-month follow-up included backward stepping response variables, forward/backward gait variables, Mini-Balance Evaluation Systems Test (MBT), and 6 Minute Walk Test.

**Results:**

There was a high retention rate (95.8%) and adherence rate (100%) with one adverse event and minimal burden. Significant improvements were seen in step count and excursion distance during backward stepping responses, forward and backward gait velocity, forward step length, and the Mini-BESTest.

**Conclusion:**

Backward cycling was a feasible intervention for people with Parkinson’s disease, demonstrating low burden with high retention and adherence rates, and it is a safe exercise with the potential for benefits in gait and balance variables.

LAY ABSTRACT

It is known that people with Parkinson’s disease often have falls due to balance issues, including an increased risk of backward falls. This may be due to known difficulties with stepping and walking in a backward direction. There is limited research looking at backward-directed exercises, therefore this study assessed the safety and effects of a backward stationary cycling program with people with Parkinson’s disease. People who participated in a 6-week backward stationary cycling program showed that it was safe, participants adhered to the program and minimal assistance was needed throughout. Also, at the end of the program, people were able to walk faster in both forward and backward directions, were able to catch themselves better following a loss of balance backward and had better balance overall. This study supports the use of this type of program in clinics.

Parkinson’s disease (PD) is a progressive, neurodegenerative disorder characterized by bradykinesia, tremors, rigidity, and postural instability (1). People with PD often experience falls, which can be detrimental to function and quality of life (2). Most falls that occur while walking tend to happen in a forward direction, possibly due to deficits in terminating gait (3). People with PD also demonstrate difficulty stepping and walking in a backward direction, and deficits in backward walking have been correlated with known predictors of falling in older adults (4–6). In standing, people with PD have a preponderance for backward falls (7–9). One study reported that 75% of falls that resulted in bone fractures in people with PD were due to backward or sideward landings, and data from fall diaries highlighted difficulties with stepping backward (9). For reactive stepping responses or compensatory steps to realign the centre of mass over the base of support following a loss of balance, people with PD tend to have a delayed and smaller response, increasing the risk of falls (10). Reactive stepping responses are commonly affected in PD due to smaller stability margins and bradykinesia (8, 9), and medications used to manage the symptoms of PD have limited effects on stepping responses (11).

Studies have assessed the effects of various exercises on backward stepping responses (BSR) and/or backward walking in people with PD, including dance, tai chi, treadmill training, and perturbation training; however in those studies, specialized equipment was needed (12, 13), a high number of repetitions of external perturbations were used (14), or exercises were led by a professional (15–17), decreasing the feasibility for use in the clinic, home, or community.

Cycling has been shown to be effective in improving physical functioning in people with PD (17). Additionally, electromyographic studies of healthy individuals have revealed similar muscle activation patterns between walking and stationary cycling (18), and similarities in lower limb muscle activation between backward cycling and BSR, in which there is greater activation of the biceps femoris, followed by greater activation of the rectus femoris (19, 20). No known studies assess a backward cycling program’s feasibility and potential effects on people with PD. Therefore, this study aimed to assess the feasibility of a 6-week backward cycling program in people with PD, specifically reporting the acceptability, suitability, and burden. The secondary aim was to report the preliminary effects of a 6-week backward cycling program on balance and gait in people with PD.

## METHODS

### Study design

This was a pre-test–post-test, single-group prospective study with a 1-month follow-up. It was approved by the Midwestern University Office of Research and Sponsored Programs (AZ 1128) and the University of Indianapolis Human Research Protections Program (reliance agreement) and conducted at the Midwestern University Physical Therapy Institute. All participants gave written informed consent prior to participation in the research study.

### Participants

Convenience sampling was used to recruit potential participants from local outpatient therapy clinics, PD exercise groups, and PD support groups. The recruitment period lasted from April 2018 to September 2019. To be included, participants needed to be aged 20 to 80 years, diagnosed with idiopathic PD, be in Hoehn and Yahr (H&Y) PD Staging Scale of stage II or III, able to walk unassisted for at least 20 feet, not enrolled in physical therapy during the study, and able to understand and speak English. Participants were excluded if they presented with PD dementia (score of less than 21/30 on the Montreal Cognitive Assessment [MoCA]) (21), were diagnosed with parkinsonism or Parkinson-plus syndrome, had the presence of another neuro-logic disorder, and/or had any injury or issue that would limit participation in any capacity. For feasibility studies, a sample size of 20–25 has been reported to be adequate; therefore, we aimed to enroll up to 28 to account for attrition (22).

### Data collection

Data were collected at baseline, post-intervention, and 1-month follow-up. Demographic and past medical history related to PD were collected, as well as BSR measures, a balance assessment using the Mini-Balance Evaluation Systems Test (MBT), a walking capacity assessment using the 6-minute walk test (6MWT), and forward and backward gait parameters. To minimize bias, all data were collected by research assistants (RAs) trained by the principal investigator (PI). The RAs were third-year Doctor of Physical Therapy students enrolled in the Midwestern University Physical Therapy Program. Interrater and intrarater reliability of the MBT was established for the raters prior to data collection. Interrater was excellent (ICC = 0.96 and 0.94), and intrarater reliability was good-to-excellent (ICC = 0.81 to 1.00).

### Procedures

*Eligibility*. Eligibility was confirmed by the MoCA and the Movement Disorders Society Unified Parkinson’s disease Rating Scale, motor subsection III (MDS-UPDRS III) by the PI. The MoCA is a validated and reliable instrument that tests various cognitive abilities (23). In addition, it can detect PD dementia with a cut-off score of less than 21/30 (21). The MDS-UPDRS III is a PD-specific comprehensive scale that assesses both motor and non-motor functions (24). Section III of the MDS-UPDRS is a motor examination and assisted the PI in determining the H&Y stage of each participant. The original H&Y scale was used; therefore, participants were labeled as H&Y stage 1 if only unilateral involvement was present; H&Y stage 2 if bilateral symptoms existed (such as resting tremor, bradykinesia, or difficulty with alternating movements) but with no postural instability (normal recovery on pull test); labeled H&Y stage 3 if the participant demonstrated postural instability (needed assistance to recover from the pull test) with bilateral symptoms; and labeled H&Y stage 4 if they presented with substantial gait impairment, moderate global bradykinesia, and postural instability (25).

*Feasibility.* Retention rates, adherence rates, and number of adverse events in the program measured acceptability and suit-ability. Retention rate was calculated by subtracting the number of participants who completed the entire 6-week intervention program from those who started the intervention. Adherence was measured by the number of participants who could complete the total intervention duration of 30 min each session. An adverse event was defined as any event that caused harm to the patient, resulting in the intervention’s discontinuation. Burden was defined as the amount of assistance participants needed during the intervention.

*Backward stepping response.* Three variables related to backward-stepping responses (BSR) were measured during the administration of the MBT subsection of reactive postural control. Time to steady (BSR-TTS) referred to the time it took from the start of the loss of balance to the moment backward momentum was stopped. Step number (BSR-StepNo) referred to how many steps were needed to steady. Maximum stepping excursion (BSR-MSE) referred to the distance needed to steady. The push and release test was essentially used to test BSR. This test has been shown to hold its sensitivity over repeated trials and has good interrater reliability (ICC = 0.84) (26). Of note, this reliability was established using an ordinal scale from 0 to 4. However, this study used a step number count for data analysis to show a change in the number of steps.

Two RAs were present for this test, and all trials were video recorded. First, the participant’s heels were lined up perpendicular to a piece of tape on the ground. Per MBT instruction protocol, the patient was asked to “stand with your feet shoulder-width apart, arms at your sides. Lean backward against my hands beyond your backward limits. When I let go, do whatever is necessary, including taking a step, to avoid a fall.” The participant remained still after steadying while a second RA placed a piece of tape at the heel of the rear-most foot. Three trials were completed. The distance between the starting heel position and the rear-most heel was measured for all 3 trials and recorded as BSR-MSE. An RA also watched the video of the trials, measured the time to steady with a stopwatch, and recorded this time as BSR-TTS. The time was started when the tester removed their hands from the shoulder blades and stopped after steadying. For BSR-StepNo, an RA counted the number of steps. The means of the 3 trials for each variable were used for statistical analysis.

*Gait analysis.* Gait parameters were collected utilizing the GAITRite^®^ system (CIR Systems, Franklin, NJ, USA), an instrumented walkway with pressure sensors that collects data on spatiotemporal gait parameters (27). It has good-to-excellent test–retest reliability (ICC = 0.79 to 0.98) (28) and excellent concurrent validity with paper-and-pencil gait analysis methods on spatial measures (right step length ICC = 0.97; left step length, ICC = 0.99) (29). Each participant walked at a comfortable pace beyond the other end of the walkway. Three trials, each of forward and backward walking, were completed, and the means were used for statistical analysis. This study used gait velocity, left and right step length, and left and right step width variables for data analysis.

### Balance

The MBT assesses 4 systems of balance: anticipatory postural adjustments, reactive postural responses, sensory orientation, and dynamic gait (30). It is a valid measure in PD when compared with other established balance measures, has excellent test–retest reliability (ICC = 0.92 to 0.98) and interrater reliability (ICC = 0.86 to 0.99), and an established minimal detectable change (MDC) score (3.5 points) (30). The test sheet includes standardized verbal instructions and set-up, therefore this protocol was followed for each participant.

*Six-minute walk test.* The 6MWT is a test of walking capacity, measuring the distance a person can walk in 6 minutes. It has excellent test–retest reliability for people with PD (ICC = 0.96) (31). A 50-foot length walkway was marked off with a cone at each end. Participants walked back and forth as far as they could in 6 minutes. Verbal instructions were used according to the recommendations of the American Thoracic Society (32) and one trial was completed.

### Intervention

Each participant completed 12 exercise sessions over 6 weeks, at a frequency of twice a week, using a Schwinn Airdyne AD6 stationary bicycle (Nautilus, Inc., Vancouver, WA, USA). This stationary bicycle was selected to provide consistent wind resistance during both forward and backward pedaling. Despite no known publications describing using an Airdyne stationary bicycle with PD, it has been used in studies with other neurologic disorders (33, 34). Attempts were made to keep the sessions consistent in terms of day and time of time; however, this was not always possible due to logistics and availability. Medication status for the intervention session was not evaluated.

All intervention sessions were carried out by an RA who was not involved in the assessments. All RAs completed a training session led by the PI prior to the study to ensure procedural consistency. The intervention sessions were held in a private examination room within an outpatient therapy clinic. During the first session, the seat height for each participant was set to allow 25° to 30° of knee flexion when the pedal was at the bottommost point. This represents the most desirable position to ease forces placed on the knee during forward cycling. Therefore, the researchers elected to use this same principle as no established literature on proper seat height exists for backward cycling (35). Participants performed a 5-minute warm-up of easy pedaling in their preferred direction, then pedaled backward at a moderate intensity for 30 minutes. Within 30 seconds of starting the intervention and every 5 minutes throughout, participants rated their intensity using a visual Borg Rating of Perceived Exertion scale (Borg RPE). The Borg RPE is a 15-point scale from 6 to 20, with 6 being the lightest intensity and 20 being the maximal intensity (36), and is a valid measure of exertion in people with PD (37). For this study, moderate intensity was defined as a rating of 12–14. If participants reported a level less than 12, they were asked to pedal faster; if above 14, they were asked to pedal slower. If a rest break was requested during the session, the timer was paused until the participant was ready to continue; therefore, all participants completed 30 min of total exercise time. After 30 minutes, a 5-minute cool-down period of easy pedaling or walking was performed. Vital signs (heart rate, respiratory rate, oxygen saturation, and blood pressure) were taken before and after each intervention session, and after exercise, the participant rested until all measures returned to within 10% of baseline.

### Statistical analysis

Data were analyzed using IBM SPSS Statistics for Windows, Version 24.0 (IBM Corp, Armonk, NY, USA). All comparisons were two-tailed, using a significance level of *p* < 0.05 to be considered statistically significant. Normal distribution of data was determined using the Shapiro–Wilk test. For comparisons, a repeated measures ANOVA for normally distributed data was used, and a Friedman ANOVA for non-normally distributed data was used. If significance was found in the repeated measures ANOVA results, post hoc tests with Bonferroni correction were run to locate the source of difference. If significance was found in the Friedman ANOVA results, post-hoc pairwise comparisons were run using the Wilcoxon signed-ranks test to locate the difference.

Post-hoc pairwise comparison effect size estimates are reported using Cohen’s *d*. For data not normally distributed (MBT, BSR-TTS, and BSR-StepNo), the z-value was used to calculate *r* proposed by Cohen to gain the effect size (38). Effect sizes for Cohen’s *d* were interpreted based on guidelines proposed by Cohen (38) with 0.20– < 0.50 = small effect; 0.50– < 0.80 = medium effect; > 0.80 = large effect. Effect sizes for *r* were interpreted as 0.10 = small effect; 0.30 = medium effect; 0.50 = large effect (39).

During testing, all falls or losses of balance that required physical assistance resulted in no data being entered for that trial, and any incomplete data sets were not included in the data analysis.

## RESULTS

**Fig. 1:**
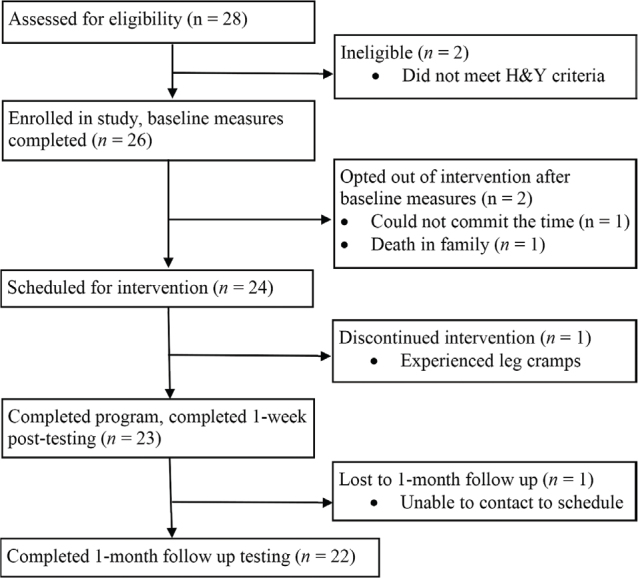
Flow of participants through study.

Twenty-six participants were eligible and enrolled in the study. Two participants were lost prior to the start of the intervention. Of the 24 who started the intervention, 23 (95.8%) were taking a form of levodopa, 2 (8.4%) had a deep brain stimulator, 2 (8.3%) demonstrated freezing during the evaluation, and 4 (16.7%) were observed with dyskinesias. One participant was lost to a 1-month follow-up; 22 participants were included in the data analysis. Participant flow is shown in Fig. 1, and baseline demographics are presented in Table I. One participant experienced a fall during backward-stepping response testing, resulting in incomplete data sets for BSR-MSE, BSR-TTS, and BSR-StepNo. Two participants had incomplete gait data due to the instrumented walkway not being able to differentiate the steps fully.

**Table I:** Baseline characteristics (*n* = 23)

Characteristic		Minimum–Maximum
Gender, *n* (%)		
Male	19 (82.6)	
Female	4 (17.4)	
Age, mean (SD)	68 (7.74)	56–78
Years since PD diagnosis, mean (SD)	6.4 (4.44)	1–14
Hoehn and Yahr stage, *n* (%)		
Stage II	11 (47.8)	
Stage III	12 (52.2)	
MDS-UPRDS III, mean (SD)	30.70 (13.10)	10–63
MoCA, mean (SD)	26.74 (2.77)	21–30

PD: Parkinson’s disease; MDS-UPDRS III: Movement Disorders Society-sponsored revision of the Unified Parkinson’s Disease Rating Scale part III motor examination; MoCA: Montreal Cognitive Assessment.

### Feasibility

*Acceptability and suitability.* Twenty-four participants were scheduled for the 6-week intervention program (see Fig. 1). Of the 24 participants who started the intervention program, 23 completed the program in its entirety, for a retention rate of 95.8%.

All participants were able to complete 30 min of total exercise time each session for an adherence rate of 100%. Within the group, 56.5% needed at least 1 rest break during the entire 6-week intervention program. For those who needed rest breaks, the total number needed for the entire 6-week program ranged from 1 to 26 (median = 1, mean = 3.22), with 2 rest breaks being the most frequently needed per session (13.0%), followed by 1 and 5 per session (8.7% each).

One adverse event was recorded: 1 participant experienced mild leg cramping in the quadriceps muscle during the second and third intervention sessions. The first experience of cramps was managed by self-massage and hydrating. However, the participant returned to the next session several minutes after starting the exercise and experienced leg cramping again. This was managed by rest and self-massage and subsided within a few minutes. The participant then opted to discontinue the study.

*Burden.* All participants were able to get on, off, and maintain a sitting balance on the bicycle without physical assistance. One participant required close supervision when on the bicycle; however, no adverse effects were noted for the duration of the study. The only modification needed for the bicycle was a gel overlay seat cover for increased comfort, which was relatively inexpensive and needed very little time to put on.

### Backward stepping response

Table II summarizes the results of the study. Post hoc analyses revealed a significant median decrease between baseline and post-intervention of 0.67 steps (*Z* = –2.00, *p* = 0.05), with a medium effect size (*r* = –0.45) and a significant median decrease of 0.83 steps from baseline to one-month follow-up (*Z* = –2.44, *p* = 0.02) with a large effect size (*r* = –0.55).

**Table II:** Comparison of baseline, post-intervention, and 1-month follow-up measures

Variable	BaseMean (SD)	Post-interventionMean (SD)	1-month follow-upMean (SD)	*p*	Effect size^b^
Backward stepping response (BSR)					
Backward stepping response–maximal stepping excursion (cm)	54.67 (25.77)	42.33 (15.53)	44.22 (13.44)	0.05	0.58
Backward stepping response–time to steady (sec)^a^	0.84 (1.31)	0.33 (0.48)	0.40 (0.77)	0.06	–0.50^c^
BSR-step number^a^	2.00 (1.25)	1.33 (1.00)	1.17 (0.92)	0.02*	–0.45^c^
Backward gait					
Gait velocity (cm/sec)	74.11 (15.63)	81.02 (18.00)	87.21 (21.71)	0.00*	0.41
Left step length (cm)	41.77 (8.22)	43.25 (9.46)	44.25 (11.19)	0.25	0.17
Right step length (cm)^a^	47.96 (12.41)	45.92 (17.26)	46.27 (16.76)	0.37	0.12
Left step width (cm)	19.47 (4.35)	18.88 (5.09)	18.43 (4.84)	0.15	0.12
Right step width (cm)	19.60 (4.63)	19.09 (4.97)	18.70 (4.70)	0.22	0.11
Forward gait					
Gait velocity (cm/sec)	112.14 (20.53)	117.83 (21.56)	119.08 (24.12)	0.05†	0.27
Left step length (cm)	63.83 (9.67)	66.27 (10.07)	66.94 (10.62)	0.01*	0.25
Right step length (cm)	64.01 (8.45)	65.97 (9.35)	66.40 (9.31)	0.04*	0.22
Left step width (cm)	9.93 (2.71)	10.42 (3.69)	10.53 (3.45)	0.22	0.15
Right step width (cm)	10.25 (2.97)	10.29 (3.62)	10.63 (3.35)	0.53	0.01
Functional outcome measures					
6 Minute Walk Test (m)^a^	369.72 (114.3)	374.45 (100.81)	369.72 (99.06)	0.24	0.20
Mini-BESTest^a^	23.00 (4.25)	25.00 (2.75)	26.00 (3.00)	0.00‡	–0.48^c^

a Reported as median (interquartile range).

b Expressed as Cohen’s *d* comparing baseline with post-test, unless otherwise stated.

c Expressed as Pearson’s *r* comparing baseline with post-test.

* *p* < 0.05.

† *p* = 0.05.

‡ *p* < 0.01.

### Backward gait parameters

Post hoc analysis revealed a statistically significant increase between baseline and 1-month follow-up, with a mean difference of 13.01 cm/s, *p* = 0.01 with a medium effect size (*d* = 0.70).

### Forward gait parameters

Post hoc analyses identified a statistically significant increase between baseline and 1-month follow-up in left step length, with a mean difference of 3.11 cm, *p* = 0.04, and a small effect size (*d* = 0.31).

### Balance

Post hoc analysis revealed a statistically significant median difference of 2 points between baseline and post-intervention (*Z* = –2.24, *p* = 0.03) with a medium effect size (*r* = –0.48) and 3 points between baseline and 1-month follow-up (*Z* = 3.50, *p* < 0.01) with a very large effect size (*r* = –0.75).

## DISCUSSION

Postural instability is common in PD with consequential fall risk, which worsens as the disease progresses (39), and postural instability in the backward direction is particularly prevalent (3, 4, 7). Exercise has been shown to improve various gait attributes in people with PD; however, there are limited studies on interventions to improve backward walking and stepping responses. The interventions that have been shown to be effective have limited usability within the home and community setting. To date, this is the first study to assess the feasibility and effects of backward cycling for individuals with PD.

All participants could pedal independently, and both the retention and adherence rate was high, supporting its feasibility within the clinic. Safety using a stationary bicycle in the PD population has been shown in the past (17) and is further supported by our study. In addition to being a safe exercise to utilize in the clinic, backward cycling may address multiple deficits, making it an efficient option in the clinic and possibly the home. Fear of falling has been implicated as a barrier to home and community exercise in people living with PD (40, 41), therefore highlighting the need for a safe and effective intervention for these environments. Additionally, other factors that may negatively affect adherence to exercise include the level of difficulty to perform the intervention, the safety of the intervention, and the location of the exercise (some preferred the home, and some preferred a gym setting) (42, 43). This intervention may be a safe and effective exercise to address PD-related functional deficits, and a stationary bicycle is a piece of equipment that can be used in either the home or clinic, and commonly found in many gym settings.

At baseline, the participants required a mean of 2.20 steps to steady from a backward loss of balance. This result is similar to a study on people with PD in H&Y stage III, which reported a mean of 2.43 steps to recover after a backward perturbation (44). After a backward cycling program, participants took statistically significantly fewer steps to recover from a backward loss of balance. This change may be relevant in the PD population, as retropulsion is a known fall risk factor (45).

Participants were able to steady themselves in a significantly shorter distance. This may be clinically relevant, as being able to steady oneself in as little distance as possible may improve safety. Although no literature exists on what constitutes the optimal distance in which one should be able to stop a loss of balance, it would seem important so as to avoid obstacles. Participants also required less time following a backward loss of balance and, while not statistically significant, potentially due to the small sample size, a large effect size was demonstrated (*r* = –0.50). This is clinically relevant as it can demonstrate quicker ability to react and stop a loss of balance, an ability affected by PD. Of note is that medication status during the study was not formally evaluated. However, research has shown that dopaminergic medications have little to no effect on postural responses and may support that the change observed in this study is actual change (11).

Backward cycling resulted in significant improvements in backward and forward gait velocity. The mean difference in backward gait velocity from base-line to 1-month follow-up was 13.10 cm/s, or 0.13 m/s. Although no MDCs have been established for backward walking speed in PD, this value approached the MDC for forward gait velocity in this population (MDC = 0.18 m/s) (30). Notably, backward gait velocity improved at a greater rate than forward gait velocity, with a baseline to 1-month follow-up mean difference for forward gait velocity of 6.94 cm/s, or 0.07 m/s. These improvements align with a previous study showing similar improvements utilizing forward recumbent cycling, in which people with PD demonstrated improved step length and gait speed (46). Backward walking is affected in persons with PD, including decreased velocity, stride length, and increased time in stance (4). Backward walking is necessary for various daily tasks, including stepping back from a closet, sink, or a dangerous situation like an approaching car (47). However, backward direction training is rarely used in physical therapy or included as part of walking intervention protocols for people with PD (48).

Backward cycling also resulted in significant improvement in balance, as measured by the MBT. The median difference of 3 points between baseline and 1-month follow-up is close to the minimal clinically important difference value of 3.4–4.0 points (49). Therefore, a backward cycling program not only shows promise in addressing backward-directed movements, such as BSR and backward walking, but can also show improvements in other important functions, including forward gait and balance strategies.

Of interest is that several variables demonstrated continued improvement from post-testing to the 1-month follow-up assessment. The authors hypothesize this may be due to participants continuing some form of continuous exercise following the 6-week program. Additionally, the learning effect of the testing measures cannot be ruled out. Additionally, backward gait velocity demonstrated significant improvements; however no significant improvements were observed with backward step length. This may be due to improvements in other variables not analyzed for this study, including increased cadence and improved stride length.

No significant changes were found in walking capacity despite participants exercising at moderate intensity. The American College of Sport Medicine Guidelines for Exercise Testing and Prescription recommends an exercise frequency of at least 3 days per week to improve or maintain aerobic capacity (50). The frequency of twice a week in the current study may not have been enough to make a significant change. Additionally, stationary cycling can significantly improve VO_2_max (a measure of aerobic capacity) without significant improvements in the 6MWT (51). Further studies are needed to explore the effects of backward cycling at a higher frequency to assess whether walking capacity improvements are observed.

Overall, a backward cycling intervention may be feasible and have positive effects on forward and backward walking, backward stepping responses, and balance. Previous studies have demonstrated improvements in stepping responses; however, they utilized equipment or procedures not feasible for the home or community, including a hydraulic platform or safety harness over a treadmill (12, 52, 53). Additionally, the ability to produce perturbations in the home setting is a significant barrier due to safety concerns and plausibility.

### Limitations

Several limitations exist within this study. The sample size was relatively small and therefore underpowered. It lacked a control group; therefore, a cause–effect of the intervention could not be established. The addition of a control group would allow comparisons with more traditional interventions. Additionally, the 6-week duration was relatively short, thus a longer duration program may have resulted in greater effect sizes. In a systematic review of 14 studies assessing PD interventions, 10 (71%) had durations of greater than 6 weeks (54). Although attempts to organize participants on a consistent schedule in terms of day and time of day, this was not consistent due to scheduling logistics and availability. Additionally, medication status during testing was not recorded, therefore variability in function due to medication status exists. The study included only those in H&Y stages II and III therefore cannot be generalized to people in other stages of the disease progression. Minimal detectable change values have not been established for backward gait using a 14-foot instrumented walkway system, therefore clinically meaningful change was unable to be determined. The study was performed under supervision in a therapy clinic and so its safety and acceptability cannot be generalized to the home. More research is needed to assess its safety in an unsupervised environment within a home setting.

### Conclusion

This study supports using a backward cycling program in the clinic as a feasible, safe intervention that may improve backward-stepping responses in people with mild to moderate PD. Backward cycling may also improve overall balance and gait parameters in both the forward and backward direction, complementing current evidence-based treatment approaches. Future research is needed to determine the optimal dosage, and to assess its safety and feasibility within an unsupervised environment to determine its use at home. Additionally, additional research is warranted to compare backward and forward cycling to assess whether one direction is superior.
